# Thermal Preconditioning Alters the Stability of Hump-Snout Whitefish (*Coregonus fluviatilis*) and Its Hybrid Form, Showing Potential for Aquaculture

**DOI:** 10.3390/biology12101348

**Published:** 2023-10-20

**Authors:** Yulia P. Sapozhnikova, Anastasia G. Koroleva, Vera M. Yakhnenko, Aleksandra A. Volkova, Tatyana N. Avezova, Olga Yu. Glyzina, Mariya V. Sakirko, Lyubov I. Tolstikova, Lyubov V. Sukhanova

**Affiliations:** Limnological Institute Siberian Branch of the Russian Academy of Sciences, 3 Ulan-Batorskaya, 664033 Irkutsk, Russia; vera@lin.irk.ru (V.M.Y.); alexa.volkova8@gmail.com (A.A.V.); fototanya@mail.ru (T.N.A.); glyzina@lin.irk.ru (O.Y.G.); sakira@lin.irk.ru (M.V.S.); lubow.tur@yandex.ru (L.I.T.); lsukhanova@yandex.ru (L.V.S.)

**Keywords:** thermal acclimation, preconditioning, aging, stress-induced senescence, telomeres, functionally active mitochondria, telomerase activity, whitefish, climate warming

## Abstract

**Simple Summary:**

Temperature fluctuations can affect the overall stability of the biomolecules involved in biological processes in ectothermic species, which can alter the overall plasticity of the species and is manifested in their accelerated cellular aging. The aim of this work was to evaluate the effects of thermal pre-adaptation/preconditioning on whitefish forms, which holds potential for aquaculture, particularly on their stress tolerance and stress-induced senescence with the use of key cellular and molecular biomarkers: the volume of functionally active mitochondria, telomerase activity, and telomere length. We concluded that thermal preconditioning appears to be highly effective in preventing telomere attrition in cold-water whitefish, and may play a preventive role in the development of stress-resistant aquaculture. The proposed biomarkers could be used at earlier stages of ontogeny as indicators of juvenile fish’s well-being and plasticity in aquaculture or the natural environment due to climate change.

**Abstract:**

One of the little-studied ways that climate warming or temperature increases in aquaculture could affect aquatic animals is through accelerated aging. This study is dedicated to understanding the principles of molecular and cellular aging in the target tissues of juvenile whitefishes (Yenisei hump-snout whitefish and its hybrid) under the influence of acute heat stress (up to 26 °C), and the effects of thermal preconditioning as pre-adaptation. Non-adapted stressed hump-snout whitefish showed a higher induction threshold for functionally active mitochondria in the blood and a decrease in telomerase activity in the liver after heat shock exposure as a long-term compensatory response to prevent telomere shortening. However, we observed heat-induced telomere shortening in non-adapted hybrids, which can be explained by a decrease in mitochondrial membrane stability and a gradual increase in energy demand, leading to a decrease in protective telomerase activity. The pre-adapted groups of hump-snout whitefish and hybrids showed a long-term or delayed response of telomerase activity to heat shock, which served as a therapeutic mechanism against telomere shortening. We concluded that the telomerase and telomere responses to thermal stress demonstrate plasticity of tolerance limits and greater stability in hump-snout whitefish compared with hybrids.

## 1. Introduction

Ambient temperature is a critical ecological feature that can act as a stressor to the ecosystem, and has attracted increasing attention in recent years. When the environmental temperature is above or below the ecological optimum, organisms may reach their physiological limits [1], and oxidative stress may occur as a result [2,3,4]. Numerous biochemical processes, including enzyme activity, membrane composition, and the stability of living organisms, can be affected by temperature changes [5].

Among the most useful, up-to-date biomarkers of past stress and stress-induced senescence are telomeres and telomerase (a ribonucleoprotein) activity [6,7,8,9,10,11,12]. Telomere repeats are very sensitive to reactive oxygen species (ROS) induced because of oxidative stress [11,13]. As a result, telomeres can shorten in the absence of elongation mechanisms, leaving the coding region of a chromosome defenseless and thus susceptible to damage, leading to senescence [14,15]. The reported rate of telomere loss is often significantly higher than if it were due to cell division alone, supporting the idea that telomere shortening also occurs under the influence of stress factors [16,17], including temperature [1,18,19,20,21], via oxidative stress pathways [22,23,24].

Moreover, adverse environmental conditions can lead to complicated physiological stress responses associated with increased rates of aerobic metabolism, production of adenosine triphosphate (ATP) by functionally active mitochondria, and consequently production of ROS [11]. ROS can damage biological substances including proteins and DNA if not quenched. It is well known that the high content of guanine in telomeric DNA makes it particularly susceptible to oxidative damage and difficult to repair [25,26,27]. Therefore, mitochondrial function (the level of functionally active mitochondria) and telomere end-cap protein status (telomerase activity) are the primary processes that directly underlie telomere dynamics [11].

Similar investigations in aquatic poikilothermic animals, such as fish, are limited [1,10,11] in comparison to the numerous studies that have addressed telomere length dynamics under oxidative stress in homeothermic mammals or birds [7,8]. However, most fish express telomerase in all tissues and at all life stages [28,29,30,31,32] and are able to extend telomeres and cell replication capacity [33]. In addition, most fish are not only poikilothermic but also indeterminately growing, so they are much more affected by ambient temperature. This is especially true for cold-water species, such as whitefishes. Specifically, a rise in water temperatures brought on by global warming has been found to lower whitefish biomass by 3–8% over a 50-year period, as shown by a mathematical model [34,35]. Thus, this work is of great importance, as whitefish stocks in some global reservoirs are currently in a critical condition [36,37,38,39].

A similar situation has recently developed for whitefishes in the Baikal-Yenisei basin. In particular, Yenisei hump-snout whitefish (*Coregonus fluviatilis* Isaczenko, 1925) [40,41,42], inhabiting Lake Baikal and previously classified as Baikal pidshian (*C. pidshian* Gmelin, 1877) [37,43,44], is currently in an extremely stressed state, to the point of complete extinction due to anthropogenic pressure [43,45].

Since whitefish fisheries in many lakes and reservoirs are declining and unable to meet market demand, the challenge of developing highly productive aquaculture of this species using knowledge-intensive technologies is particularly urgent [43]. The hybrid of Yenisei hump-snout whitefish with Baikal whitefish (*C. baicalensis* Dybowski, 1874) [40,41] are also considered as promising from the perspective of commercial production [43]. In particular, the hybrids obtained by artificial insemination (included the use of cryopreserved sperm) showed a good growth rate in aquaculture conditions and can be considered as promising for further cultivation, although there are no data on the stress resistance and stability of these forms. In this case, we mean man-made hybridization, since wild natural hybrids of Yenisei hump-snout whitefish with Baikal whitefish have not been found due to high or complete separation of spawning periods and complete separation of spawning areas [46] (see Section 2.1). However, even artificial hybrids, whose existence is associated only with man-made hybridization, may soon become ubiquitous in the natural environment due to shifting climatic conditions. That is why understanding their physiological capacity, in particular under the influence of critical temperature, is important.

Whitefish juveniles, which are primarily cold-water fish, grow best in water between 13 and 18 °C [5,34,36,47,48,49]. Higher temperatures above 22 °C slow growth [34,35,49,50], while temperatures above 26 °C are fatal to a number of whitefish species [34,49,50,51,52]. In particular, the physiological, biochemical, and immunological conditions of fish are adversely affected by extreme temperature drops of 6–9 °C, typical of cold-water whitefish aquaculture. Moreover, temperature jumps of up to 26 °C (room temperature) are very common when cooling systems fail or during fish transport [34,50,53]. This leads to temperature stress and eventually to heat shock [53].

We see a promising solution for stress-resistant aquaculture development in preliminary acclimation through “thermal training”, or “thermal preconditioning”. Thermal preconditioning, or acclimation to long-term temperature fluctuations, allows organisms to maintain their physiological efficiency in response to shifting environmental factors [34,47]. Acclimation occurs by switching enzymes and other biochemical systems to different temperature optima, altering tolerance ranges to prevailing environmental conditions, extending the limits of thermopreferendum (more common in riverine species), and enabling survival and successful existence under adverse thermal conditions in the environment [54,55,56]. Acclimation is also associated with the concept of “hormesis”, which can be used as a quantitative measure of biological plasticity, where exposure to high stressors is inhibitory, while low (mild and sub-lethal) doses are stimulatory [54,55,56]. Taken together, these studies show that the thermal history and adaptive plasticity of species are interrelated, and that the induction threshold of this adaptation can shift in response to thermal history (thermal preconditioning) [47,57].

Accordingly, in this work we test the hypothesis of a positive effect of thermal preconditioning on whitefish juveniles. In modeling the effects of thermal preconditioning and its influence on subsequent adverse thermal conditions, the following fish forms were included in the experimental scheme: (1) potentially plastic wild species, such as Yenisei hump-snout whitefish, living within very wide limits of the thermopreferendum in a niche with the most severe temperature fluctuations (in the coastal zone, at a shallow depth of ~20 m) [43,46], and (2) artificially obtained hybrid forms that are promising for aquaculture, such as hybrids of hump-snout whitefish with Baikal whitefish, which are expected to have wider limits of the reaction norm due to the possible effect of heterosis.

The complex analysis used in this work includes a number of tools to assess thermal stress and its consequences at the cellular and molecular levels: a pool of functionally active mitochondria, telomerase activity, and the relative length of telomeres. An additional biomarker of oxidative stress that has been used in this work is the leukocyte “profile” (also called “complete blood count”, “leukocyte differential”, or “hemogram”), which is considered as one of the most sensitive indicators of an adequate stress response in fish [58,59].

## 2. Materials and Methods

### 2.1. Fertilization, Incubation, and Rearing of Juveniles

The experiment was carried out on 5-month-old juveniles of Yenisei hump-snout whitefish and its hybrid (F1) with Baikal whitefish (♀ Baikal whitefish, Chivyrkuisky population, native caviar × ♂ Yenisei hump-snout whitefish, Barguzinsky population, cryopreserved sperm). The sampling of hump-snout whitefish eggs and their fertilization for subsequent incubation of non-hybrid offspring were carried out in October 2021, 80 km above the village of Novy Uayan (the Republic of Buryatia, the Upper Angara River, 56°10′50.1″ N 111°37′56.5″ E). Cryopreservation of hump-snout whitefish sperm was carried out in the same place in October 2021.

The presence of cryopreserved sperm made it possible to obtain hybrids between individuals belonging to species that are completely separated in terms of spawning periods and geographical location of spawning grounds. Baikal whitefish, unlike Yenisei hump-snout whitefish, spawn not in tributaries (September–October), but in the lake itself—in the shallow waters of bays and straits, in November–December [46]. Obtaining native Baikal whitefish eggs and performing experiments to produce F1 hybrids were carried out at the end of December 2021 in Chivyrkuisky Bay on Lake Baikal in the Baikal whitefish spawning migration areas (Republic of Buryatia, area of the settlement of Kurbulik, 53°42′14.3″ N 109°02′16.8″ E).

The subsequent incubation of fertilized eggs was carried out in the settlement of Listvyanka (Baikal museum SB RAS) using a system of reduced copies of Weiss apparatuses, used for incubating whitefish eggs on a production scale, with aerated running Baikal water, where they were incubated at a temperature of 3–6 °C until hatching (April 2022). The subsequent methods for keeping larvae (at 10–12 °C) and juveniles (at 15–17 °C) of hump-snout whitefish and first-generation (F1) hybrids of this species with Baikal whitefish were developed on the basis of the Experimental Freshwater Aquarium Complex for Baikal Hydrobionts at Limnological Institute (LIN SB RAS). All work on artificial fertilization and further development of fertilized eggs were carried out in accordance with recommendations drawn up on the basis of many years of experience in organizing temporary points for collecting and storing eggs, physiological and embryological studies and data characterizing the spawning conditions of whitefishes, and morphological and physiological indicators of egg development [60].

### 2.2. Experimental Design and Tissue Collection

**Figure 1:**
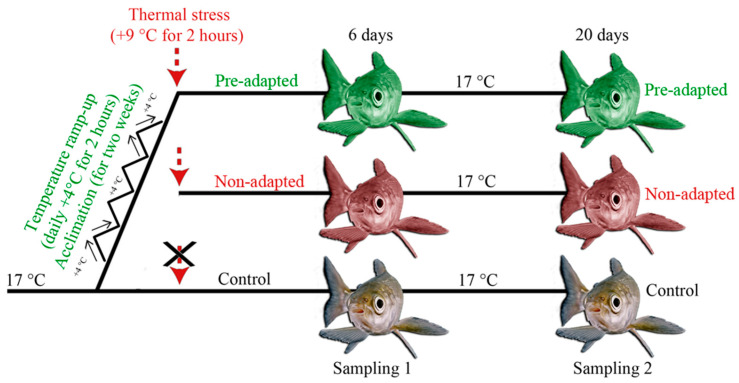
The experimental set-up (identical for hump-snout whitefish and its hybrids). Pre-adapted fish were exposed to a daily temperature increase in advance (+4 °C relative to the control for 2 h). Next, the water temperature in the experimental “acute temperature rise” aquariums with pre-adapted and non-adapted fish was increased up to 26 °C (+9 °C compared to the control for 2 h).

The experimental design was as follows: a slight increase and subsequent decrease in temperature was made during thermal preconditioning in 50-liter fish aquariums (+4 °C relative to a control temperature of 17 °C) on further exposure to lethal heat shock (+9 °C relative to a control temperature of 17 °C, up to 26 °C) (Figure 1). The total duration of preconditioning training was two weeks (a daily one-time increase of temperature by 4 °C for 2 h compared to the control, followed by a decrease to baseline values).

The temperature in the control aquariums was kept constant at 17 °C throughout the experiment. In the experimental aquariums, water was heated using a Heater 009 (Barbus, Russia, Elektrostal) aquarium heater (50 W) with the use of a temperature control sensor. To ensure that the whole aquarium (50 liters each) was at the same temperature and sufficient oxygen level, air pumps with air stones were used.

Control individuals and individuals exposed to lethal temperature were considered in the experiment (this group included two subgroups: with preconditioning training (pre-adapted fish) and without (non-adapted fish)). Sampling was carried out on day 6 (Sampling 1, short-term effect) and day 20 (Sampling 2, delayed effect) after exposure to lethal temperature (Figure 1). The time frame used in this work has been shown to be effective for assessing other stressors in previous studies [61,62].

A total of 112 5-month-old whitefish juveniles were analyzed (Table 1). For each experimental and control group, 7–9 individuals were selected, which is sufficient for the types of analysis being conducted. For the analysis of telomere length and telomerase activity, the tissues considered to respond adequately to thermal stress were selected: the liver and the gills. To assess the immune response, smears of peripheral blood and major hematopoietic organs (the kidneys and spleen) were taken, which were then examined microscopically. Selected tissues were analyzed from each selected fish using all the types of analyses listed: telomere analysis in the liver and the gills; telomerase activity assessment in the liver and the gills; samples to assess the level of functionally active mitochondria in the blood; smears of blood, spleen, and kidneys. Respectively, a total of 896 samples were collected (Table 1). Despite the fact that for each group the number of fish was sufficient, each sample was analyzed twice to exclude methodological artifacts.

The dissolved oxygen and carbon dioxide concentrations were measured in all aquariums. The iodometric method (Winkler method) was used to determine dissolved oxygen, and the detection limit was 0.2 mg/L [63]. Carbon dioxide concentration was measured by titration of the samples with sodium carbonate in the presence of the indicator phenolphthalein, and the detection limit was 0.6 mg/L. The data is presented in Table 2.

### 2.3. Ethical Standards

On each day of sampling, on the 6th and 20th day after the start of exposure (Figure 1), fish from the control and experimental aquariums were collected with a dip net. Whitefish from each aquarium were euthanized with tricaine mesylate (MS222) according to the AVMA Guidelines for Euthanasia of Animals, 2020. The samples were collected from the euthanized fish. The experiments and tissue collection were performed in accordance with the animal welfare laws, guidelines, and policies of Russia, and approved by the Ethics Committee of the Limnological Institute SB RAS.

### 2.4. Cytometric Parameters of Peripheral Blood and Hematopoietic Organs

Using techniques previously tested in other animal species, we calculated the mitochondrial membrane potential of red blood cells as a measure of cell condition [61,62,64]. In summary, the red blood cells were incubated in Medium 199 with MitoTracker Orange (100–500 nM, Life Technologies, Waltham, MA, USA) and Hank’s salts (PanEko, Moscow, Russia, Cat. No. S230p) at 37 °C for 25 min. Cells were then fixed for 15 min with 2% paraformaldehyde. DAPI solution (10 μg/mL in PBS) (Sigma-Aldrich, Burlington, MA, USA, Cat. No. D9542) was used to stain the nuclei for 15 min. Then, the preparations were coverslipped with ProLong Gold Antifade Reagent (Life Technologies, Waltham, MA, USA) and explored using an LSM 710 laser confocal microscope (Zeiss, Oberkochen, Germany). Two programs were used to process the confocal images: Imaris Bitplane version 7.2.3 and ZEN 2010 (Zeiss). Layer by layer (2D slices), as Z-stack volumes, the red blood cells represented were examined. We divided the Z-stacks into smaller fragments and found the volumes that the MitoTracker Orange fluorescent marker occupied using the Imaris 7.2.3 Bitplane AG software program (https://imaris.oxinst.com/, accessed on 16 October 2023). The entire array of fluorescent signals in the chosen region was highlighted, and their volumes were then automatically added together. After converting the overall volume of mitochondria to the relative volume of all fragments, the volume was recalculated to 1 × 10^6^ μm^3^ (100 μm × 100 μm).

In order to detect inflammatory processes and signs of oxidative stress, azure eosin-stained smears of the peripheral blood and hematopoietic organs (the kidneys and spleen) from both hump-snout whitefish and hybrids were analyzed using an Axiostar plus light microscope fitted with an AxioCam ICc1 camera (Zeiss, Jena, Germany). Hemograms were measured in the kidney and spleen (percent ratio of blasts, phagocytes, lymphocytes, and plasmocytes) and the peripheral blood (percent ratio of erythroblasts, mature red blood cells, lymphocytes, monocytes, and neutrophils). Between 139 and 394 blood cells were analyzed for each sample. Image-Pro Plus 6.0 software (https://mediacy.com/image-pro/, accessed on 16 October 2023) was used to measure the size of the cells. The formula for the area of an ellipse was used to compute the cell area (S), nuclear area (s), and nuclear-to-cytoplasmic ratio (NCR) of red blood cells. Another quantitative measure of red blood cell shape was numerical eccentricity (E) [61,62,65].

### 2.5. Telomere Length and Telomerase Activity Analysis

The polymerase chain reaction (qPCR) was used to measure telomere length. Using the phenol/chloroform method, genomic DNA from each fish (6th and 20th day) was extracted from the liver and gills of control and experimental fish [66,67]. Using Rotor-Gene Q 6000 (QIAGEN, Hilden, Germany) and the qPCR method, the relative telomere length (RTL; telomere DNA concentration/single-copy gene concentration, T/S) was determined according to Cawthon [68]. The reference gene was the glyceraldehyde-3-phosphate dehydrogenase (GAPDH) gene. A primer pair was constructed using the Atlantic salmon gene sequence (BT045621). The primer sequences were ACAGCCTACGACAGAGACTAA (reverse) and GCACTCACACCCTCCATAAC (forward). A total of 0.25 mM dNTPs, 0.2 U Snp-polymerase, 2.5 mM MgCl2 (Evrogen, Moscow, Russia), 1× Snp-buffer, 0.5-fold SYBR Green (Lumiprobe, Hunt Valley, MD, USA), 0.2–0.3 ng DNA, and 0.5 pmol of each GAPDH primer were used in the qPCR mix. For the qPCR analysis of telomere repeats, instead of adding the GAPDH primers to the reaction mixture, 0.17 pmol of Tel1 and 0.5 pmol of Tel2 primers were used. For 3 min, DNA polymerase was activated at 95 °C. The telomere reaction was immediately run for 45 cycles of 15 s at 95 °C and 2 min at 54 °C. The reference gene fragment was amplified using touchdown qPCR, which involved progressively lowering the primer annealing temperature from 64 to 58 °C over the course of the first seven cycles. One cycle for GAPDH comprised the following stages: 10 s at 95 °C, 15 s at 58–64 °C, and 15 s at 72 °C. The cycle was repeated 35 times. Using qPCR, telomere length was measured three times for each sample. Mean ± SD was used to represent T/S values.

Next, we measured telomerase activity using a real-time telomere repeat amplification protocol (Q-TRAP) [61,62] in order to supplement the telomere length measurements. Using CHAPS buffer, the total protein was extracted from the liver and gills of control and experimental fish as mentioned in Yip et al. [69]. We used acetonitrile (Cryochrom, St. Petersburg, Russia) to purify the protein mixture from the CHAPS buffer components for the more accurate concentration measurement. A total of 200 µL of acetonitrile was added to 50 µL of protein mixture and centrifuged at 13.4× *g* for 10 min. After dissolving the sediment in 8 M urea (Sigma-Aldrich, Burlington, MA, USA), the protein concentration was measured using a commercial assay (Sileks, Moscow, Russia) with the Bradford technique [70]. The Rotor-Gene Q 6000 instrument (QIAGEN, Hilden, Germany) was used to carry out the real-time Q-TRAP assay as described in Yip et al. [69] with minor adjustments. One-fold buffer (Evrogen, Moscow, Russia), one-fold Encyclo DNA polymerase, 0.25 mM dNTPs, 0.5-fold SYBR Green, 1 pmol telomerase substrate (TS) primer [71], 0.5 pmol ACX primer [72], and 200 ng of protein mixture were contained in 15 µL of the resultant solution. The procedure started with TS primer extension by telomerase being incubated for 30 min at 14–17 °C (matching the habitat of whitefish). This was followed by an incubation period of 10 min at 95 °C to deactivate telomerase and activate Encyclo polymerase. The following conditions were applied to the samples for 35 PCR cycles: 30 s at 95 °C, 30 s at 58 °C, and 1 min at 72 °C. As a no-template control, a lysate-free control (containing every component except the protein) was used. Every sample was tested twice. The semi-logarithmic amplification plots, which show the logarithmic growth in the fluorescence signal versus the cycle number, were used to calculate the cycle thresholds. Using Rotor Gene software version 2.3.1, the ΔΔCt method [73] was used to calculate the relative telomerase activity (RTA). The telomerase activity of the first control fish was set at 1, and the subsequent data were computed using the chosen control as a reference [69].

### 2.6. Statistical Analysis

The homogeneity of variances (Breakdown and 1-way ANOVA, Brown–Forsythe test) and normality of samples (Shapiro–Wilk test) were pretested. Although all of the samples exhibited distributions with similar variance (*p* > 0.05), some of them did not follow the law of normal distribution. Thus, we used non-parametric statistics for reliability. The differences between the control and experiment in all tissues for blood parameters, telomere length, and telomerase activity analysis were estimated using a Kruskal–Wallis test (Statistica 10 software package). The results were considered statistically significant at *p* < 0.05.

## 3. Results

### 3.1. Peripheral Blood Profiles

**Figure 2:**
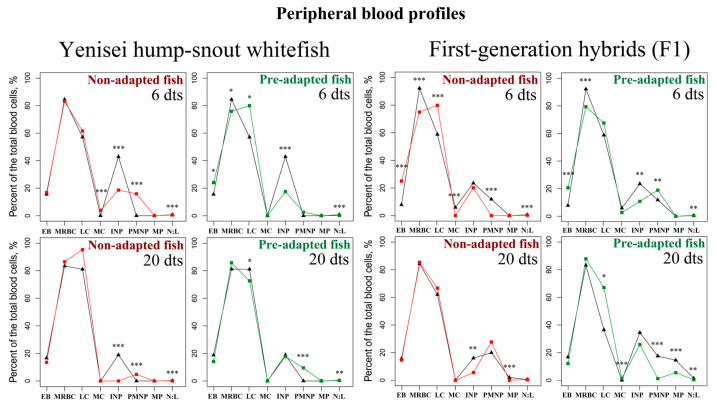
Comparison of whitefish peripheral blood profiles (percentage of the total blood cells) in control ∆ (black lines) and following 6 and 20 days of thermal exposure □ (red lines in non-adapted and green lines in pre-adapted): Red blood cells: EB—Erythroblasts, MRBC—Mature red blood cells; White blood cells: LC—Lymphocytes, MC—Monocytes, INP—Immature neutrophils, PMNP—Polymorphonuclear neutrophils, MP—Macrophages, N:L—Neutrophil to lymphocyte ratio. The total lymphoblasts and lymphocytes are included in lymphocytes. dts—days after thermal stress. Asterisks denote statistically significant differences between the experimental and control groups: * *p* < 0.05, ** *p* < 0.01, *** *p* < 0.001 (Kruskal–Wallis test). Data indicating standard deviations (SD) are given in Table A1.

Thermal exposure led to changes in the peripheral blood profiles in hump-snout whitefish and its hybrid. The main results are presented in Figure 2 (see Table A1 in Appendix A for more detailed information). Below we highlight the most important points.

First of all, in fish exposed to temperature shock, a significant (*p* < 0.01) decrease in immature neutrophils (INP) in the peripheral blood was observed: short-term, only on day 6, mostly in both groups of hump-snout whitefish and in the pre-adapted hybrid; and long-term, up to 20 days, in the non-adapted groups (see Figure 2). We also noted a significant (*p* < 0.01) increase in the level of polymorphonuclear neutrophils (PMNP) in hump-snout whitefish after temperature stress: long-term, 6 and 20 days, in non-adapted fish and with a delayed effect, up to day 20, in pre-adapted fish. On the contrary, in the non-adapted hybrid, there was a decrease in this parameter already on day 6 (see Figure 2).

In the pre-adapted hump-snout whitefish and both groups of hybrids, we also noted a significantly (*p* < 0.05) increased content of lymphocytes (LC) in the peripheral blood profiles (see Figure 2). However, only pre-adapted hump-snout whitefish showed a significant (*p* < 0.05) decrease in this indicator relative to the control on day 20. Accordingly, only pre-adapted hump-snout whitefish had a high neutrophil to lymphocyte ratio (N:L ratio) in the peripheral blood samples on day 20 (*p* < 0.01) (Figure 2, Table A1). Changes in the erythrocyte nuclear cytoplasmic ratio (NCR), which characterizes the level of transcription, were also significantly greater in hump-snout whitefish (Table A2) (see Table A2 in Appendix A for more detailed information).

Finally, it is interesting to note that non-adapted hump-snout whitefish showed significantly (*p* < 0.001) increased levels of monocytes (MC) in the peripheral blood in response to a stressful situation, while in non-adapted hybrid this parameter significantly (*p* < 0.001) decreased after thermal exposure (Figure 2). It is also noteworthy that hybrid responded to stress by a short-term change, on day 6, in the ratio of mature red blood cells (MRBC) and erythroblasts (EB) in the peripheral blood profiles (see Figure 2).

### 3.2. Blood Profiles in the Kidneys and Spleen

**Figure 3:**
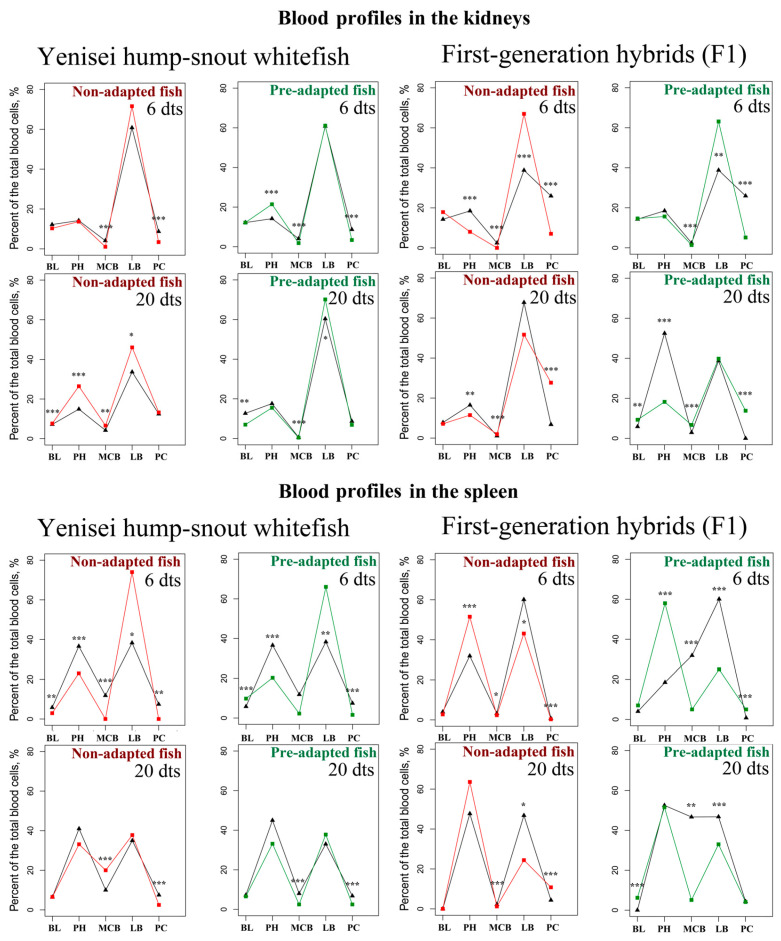
Comparison of whitefish blood profiles in the hematopoietic organs (the kidneys and spleen) (percentage of the total blood cells) in the control ∆ (black lines) and following 6 and 20 days of thermal exposure □ (red lines in non-adapted and green lines in pre-adapted): BL—Blasts, PH—Phagocytes, MCB—Monocytoblasts, LB—Lymphoblasts, PC—Plasmocytes. dts—days after thermal stress. Asterisks denote statistically significant differences between the experimental and control groups: * *p* < 0.05, ** *p* < 0.01, *** *p* < 0.001 (Kruskal–Wallis test). Data indicating standard deviations (SD) are given in Table A3 and Table A4.

The main results are presented in Figure 3 (see Table A3 and Table A4 in Appendix A for more detailed information). Below we emphasize the most important points.

In general, under the influence of heat stress, the monocytoblasts (MCB) and plasmocytes (PC) significantly (*p* < 0.001) decreased in the kidneys in all experimental groups on day 6, both in hump-snout whitefish and its hybrid. Then, on day 20, we observed a significant (*p* < 0.01) increase for monocytoblasts (MCB) in all groups and for plasmocytes (PC) in hybrids (Figure 3).

Another interesting fact is a significant (*p* < 0.001) increase in the level of phagocytes (PH) in the kidneys of hump-snout whitefish, in particular, on day 6 for the pre-adapted group (Figure 3). Meanwhile, in hybrids, the phagocytes (PH) were significantly (*p* < 0.01) reduced in response to stress in the kidneys, especially in the non-adapted hybrids with a long-term response, on day 20.

In addition, a significant (*p* < 0.05) increase in the lymphoblasts (LB) was noted in the kidneys in hybrids on day 6 and in hump-snout whitefish on day 20 (Figure 3). On the contrary, in the spleen, the reaction of this type of cell differed between hybrids and hump-snout whitefish: a long-term decrease in hybrids (*p* < 0.05) and increase in pre-adapted and non-adapted hump-snout whitefish (*p* < 0.05) on day 6, with a subsequent decrease (see Figure 3). Finally, we noted the active formation of the blasts (BL) in the spleen of the pre-adapted hump-snout whitefish and its hybrid (Figure 3).

### 3.3. Functionally Active Mitochondria

**Figure 4:**
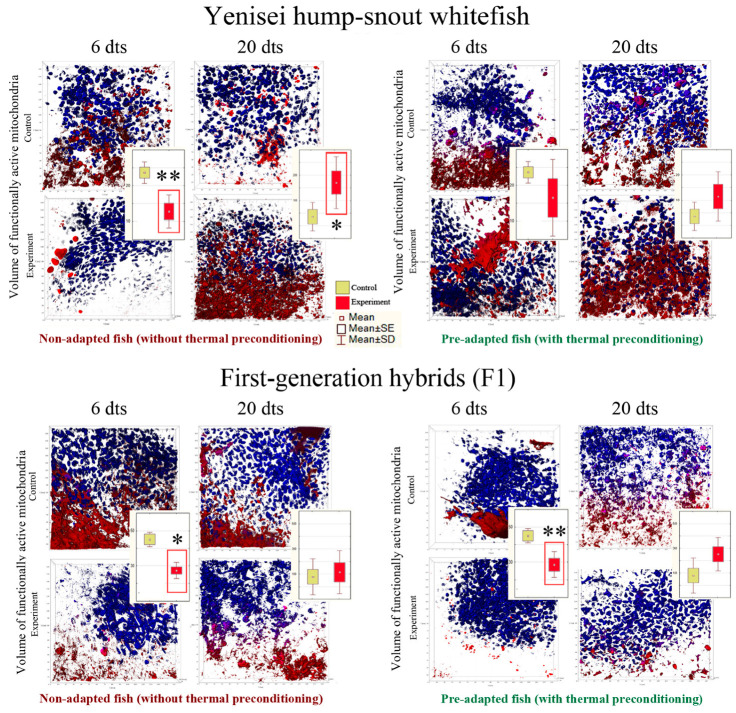
Functionally active mitochondria with a preserved membrane potential: staining for native mitochondria (MitoTracker Orange CMTMRos, red) and nuclei (DAPI, blue). N = 7–9 fish per group. dts – days after thermal stress. Asterisks and red boxes denote statistically significant differences between the experimental and control groups: * *p* < 0.05, ** *p* < 0.01 (Kruskal–Wallis test).

The main results are presented in Figure 4 (red boxes denote statistically significant differences between the experimental and control groups). Below we highlight the most important points.

Examination of the volume of functionally active mitochondria (FAM) in erythrocytes using the MitoTracker label revealed that the pre-adapted hump-snout whitefish did not show any significant decrease in FAM from the controls (Figure 4). However, significant differences were observed between non-adapted individuals and control fish at both 6 days after exposure to lethal temperature (*p* = 0.008) and after 20 days of exposure (*p* = 0.049). At day 6 post-exposure, the volume of functionally active mitochondria was reduced by 54% on average in non-adapted hump-snout whitefish compared to controls (Figure 4). At the same time, an adaptive enhancement or the so-called “enriched pool” response in the relative volume of active mitochondria (by 79% on average) was observed in the erythrocytes of non-adapted hump-snout whitefish on day 20 after the recovery period (Figure 4).

### 3.4. Telomerase Activity

**Figure 5:**
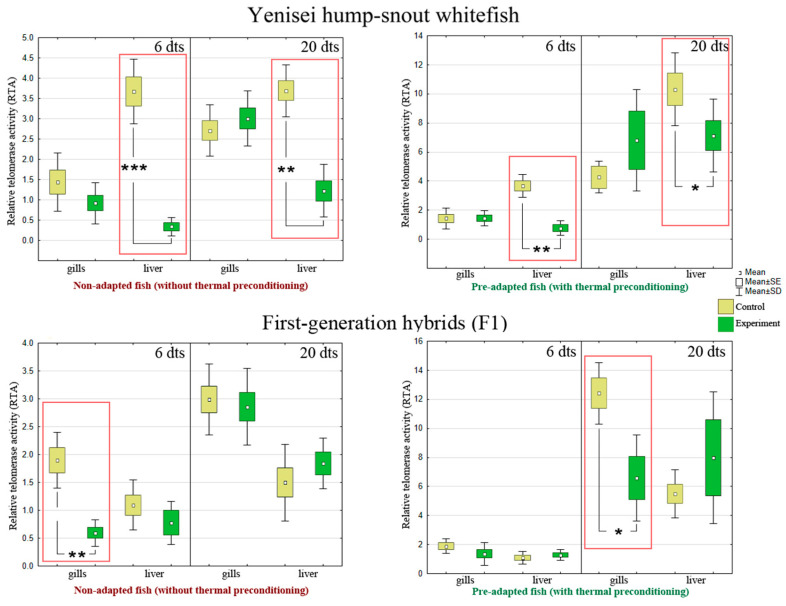
Telomerase activity levels (N = 7–9 fish per group). SE is the error of the mean; SD is the standard deviation from the mean. dts—days after thermal stress. Asterisks and red boxes denote statistically significant differences between the experimental and control groups: * *p* < 0.05, ** *p* < 0.01, *** *p* < 0.001 (Kruskal–Wallis test). Data indicating standard deviations (SD) are given in Table A5.

The main results are presented in Figure 5 (red boxes denote statistically significant differences between the experimental and control groups) (see Table A5 in Appendix A for more detailed information). Below we highlight the most important points.

At the level of telomerase activity, we observed a significant decrease in the response in the liver of hump-snout whitefish as early as 6 days after exposure to lethal temperature, in both pre-adapted (*p* = 0.009) and non-adapted individuals (*p* = 0.0006) (Figure 5).

On day 20 of the experiment, we also observe a significant decrease in telomerase activity in the liver in pre-adapted (*p* = 0.055) and non-adapted hump-snout whitefish (*p* = 0.0012) (Figure 5).

A decrease in telomerase activity in hybrids was observed at the gill tissue level in the early stages of the experiment—after 6 days, but only in individuals that were not pre-adapted (*p* = 0.006) (Figure 5). Nevertheless, at later stages of the experiment—after 20 days—we observed a decrease in telomerase activity even in pre-adapted individuals at the gill level (*p* = 0.043) (Figure 5).

### 3.5. Telomere Maintenance

**Figure 6:**
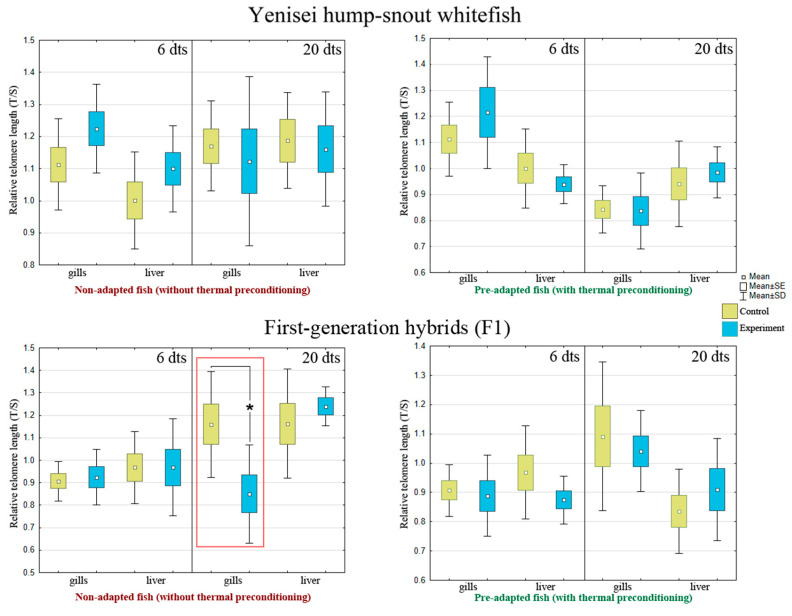
Relative telomere length (N = 7–9 fish per group). SE is the error of the mean; SD is the standard deviation from the mean. dts—days after thermal stress. An asterisk and the red box denote statistically significant differences between the experimental and control groups: * *p* < 0.05 (Kruskal–Wallis test). Data indicating standard deviations (SD) are given in Table A6.

The main results are presented in Figure 6 (red boxes denote statistically significant differences between the experimental and control groups) (see Table A6 in Appendix A for more detailed information).

When comparing the relative length of telomeres in gill tissue and liver throughout the experiment, significant differences were found only in the non-adapted hybrids in gill tissue (*p* = 0.035) (Figure 6).

Notably, the individuals that were not preconditioned showed a significant decrease in telomere length when exposed to lethal temperature, while telomere length did not decrease in the pre-adapted hump-snout whitefish and hybrids when exposed to lethal temperature (Figure 6).

## 4. Discussion

### 4.1. Cellular Blood Reactions

In general, acute thermal stress induced an adequate increase in the percentage of mature neutrophils (neutrophilia) or lymphocytes (lymphocytosis) and a reduction in the immature neutrophils in the blood of the experimental fish. The shifts in the profile of mature blood cells in pre-adapted hump-snout whitefish were consistent with a rise in the content of immature blasts in the hematopoietic organs, the kidneys and spleen, which shows an increase in erythropoiesis processes [59]. The changes listed above are thought to ensure that blasts are directed to the tissues where they are needed during the stress response [59].

However, in pre-adapted hump-snout whitefish, we observed a high ratio of neutrophils to lymphocytes (N:L ratio, neutrophilia, and lymphopenia), which was apparently due to the redistribution of lymphocytes from the blood to other tissues. This response has been previously investigated in some fish species and suggested as a substitute technique for evaluating glucocorticoids that activate the immune system and generally cause all systems to function maximally [59,74,75,76,77]. Neutrophils are the primary phagocytic leukocytes and proliferate in the bloodstream in response to inflammation and stress [59,78,79]. Numerous immunological processes, including the synthesis of immunoglobulins and the control of the immune response, are carried out by lymphocytes [80].

At the same time, an increase in phagocytes in the kidneys, both in pre-adapted and non-adapted hump-snout whitefish, indicates that functionally mature cells retain high phagocytic activity as a response to thermal exposure. Thus, the immune system of non-adapted hump-snout whitefish also copes well with a stressful situation and, as a result, we can observe an increase in the neutrophil count in the peripheral blood (stress-induced neutrophilia).

In contrast, non-adapted hybrids show symptoms of immunosuppression. In particular, this manifests in a long-term decrease of the lymphoblasts in the spleen, the phagocytes in the kidneys, the functionally active neutrophils and the monocytes in the peripheral blood. This fact can be associated with the activation of myelopoiesis [59,81] and the formation of a large number of young cells with reduced functional activity (Figure 3). This fact is also confirmed by a significant decrease in the nuclear–cytoplasmic ratio and nuclear area of red blood cells in non-adapted hybrids (Table A2), compared with the pre-adapted ones, indicating a decline in the level of transcription after thermal stress. In non-adapted hybrids, it is the immunosuppressive effect caused by the influence of stress-inducing hormones that apparently also explains the decrease in the number and activity of inflammatory cells in the blood after modeling acute thermal stress. Against this background, an increase in phagocytic function in the spleen was observed in non-adapted hybrids (Figure 3).

In summary, examination of the hemograms revealed that an adequate short-term (up to 6 days) immune response was observed in both hump-snout whitefish and hybrids that have been adapted before exposure to thermal stress, whereas a persistent immunosuppressive response (up to 20 days) was shown in non-adapted hybrids after thermal stress. Previous studies [76] have indicated that among the listed effects of acute inflammation is a defensive response that distributes plasma proteins to the injured sites in the tissues while inducing neutrophil infiltration. Thus, these cellular immune responses listed above, in conjunction with increased oxidative stress resulting from inflammation [76,78,81], were apparently the impetus for the development of subsequent responses at the level of mitochondria, telomerase, and telomeres in hump-snout whitefish and hybrids (see Section 4.2).

### 4.2. Effects of Heat Shock on Mitochondria, Telomerase Activity, and Telomere Attrition

In this section, we summarize the results obtained on different biomarkers of aging. The short-term decrease in the volume of functionally active mitochondria (FAM) on the 6th day in non-adapted hump-snout whitefish and hybrids and in pre-adapted hybrids (see Figure 4) can be explained by a change in energy consumption during the depletion of organism resources after thermal stress. The functional recalibration linked to the pathophysiology of stress is caused by these mitochondrial alterations, and is referred to as the mitochondrial allostatic load [61,62,82]. Indeed, mitochondria are thermally sensitive, and critical thermal aspects set limits for the production of reactive oxygen species (ROS) [11,83,84]. Exposure to temperatures above normal may alter the integrity of the mitochondrial membrane, raising the ratio of mitochondrial ROS/ATP production and decreasing the capacity for ATP synthesis [85], encouraging the release of cytochrome c [85], and causing subsequent oxidative damage. In addition, polyunsaturated fatty acids can also be oxidized in the membrane, with free radical cascades rapidly damaging non-adapted mitochondria and other organelles [82,86,87].

In the experiments in non-adapted hump-snout whitefish exposed to high temperature, an increase in the relative volume of functionally active mitochondria (Figure 4, day 20) can be associated with the term “enriched pool” [61,62,64]. This effect may be related to an increase in cellular energy expenditure during the mobilization of the organism’s energy reserves, and suggests the existence of natural mechanisms in hump-snout whitefish for recovery after temperature stress (Figure 4). Due to the available energy potential of mitochondria involved in the stress response, telomerase continues to react actively, so telomere length is not shortened (Figure 6). Apparently, in this case, we observed a reverse response of telomerase to form a spare capacity of mitochondrial respiration for the unanticipated energy demand of cells in a stress situation. This substantial increase in the number of functionally active mitochondria in hump-snout whitefish may indicate a possible defense mechanism against replicative senescence triggered by stress.

In turn, the pre-adapted hump-snout whitefish after thermal exposure shows no significant changes in the FAM level (Figure 4) compared to the non-adapted hump-snout whitefish (Figure 4). This could be due to the fact that mitochondrial function may change due to membrane fluidity and remodeling of these membranes during preconditioning (homeoviscous adaptation) [84,88], as previously suggested when studying the effects of natural acclimation across different seasons [89]. In particular, abalone (genus Haliotis) and tube worms (*Riftia pachyptila*) exhibit enhanced mitochondrial membrane fluidity in response to brief temperature changes, which correlates with changes in the respiratory capacity of mitochondria under the influence of temperature [84,90,91].

Previous observations also show that several compounds affect mitochondrial resistance, including the telomerase protein, which provides protection against stress-induced mitochondrial loading [11,89,92,93]. Specifically, telomerase can protect cells from damage by ROS by binding to mitochondrial DNA and mitochondrial transfer RNA, since reverse transcriptase is frequently located in mitochondria, whereas telomerase RNA is often found in the cytoplasm [94,95,96]. However, mitochondrial function and telomere maintenance have previously been considered to be closely linked [11,94], and mitochondrial dysfunction results in telomere shortening and foci in which DNA damage response factors are recruited to uncapped telomeres [94,97]. These findings imply that telomerase may play an important non-canonical role in cell survival and aging by protecting mitochondria [98].

In our experiments, we have studied telomerase as a potential therapeutic target for heat shock in whitefishes. According to the results obtained, a decrease in telomerase activity is observed in non-adapted and pre-adapted whitefishes in the liver and gills (Figure 5) in the absence of telomere shortening in hump-snout whitefish (Figure 6) after thermal stress, which can be considered a compensatory species-specific mechanism to maintain the adequate telomere length.

In the pre-adapted hybrids, the telomerase response is delayed, up to day 20 (Figure 5), but is sufficient for feedback with mitochondria and maintenance of energy resources and prevention of senescence (Figure 6). Such a response in the pre-adapted hybrids after thermal stress is likely due to remodeling of the membranes during preconditioning, resulting in saving of energy resources to maintain homeostasis.

However, as evolutionarily novel and unstable forms, non-adapted hybrids, obtained by artificial hybridization using cryopreserved sperm (see Section 2.1), show telomere shortening due to depletion of mitochondrial energy resources and telomerase activity (Figure 6, red box). It seems that in non-adapted hybrids, the bidirectional “mitochondria–telomerase” feedforward loop does not function well, since the telomerase response in gills is short-term, up to 6 days (Figure 5). Additionally, non-adapted damaged mitochondria produce more ROS [11,99], leading to physiological aging on day 20 and reflecting in telomere length shortening in response to thermal stress in non-adapted hybrids (Figure 6, red box). In addition, it is in non-adapted hybrids that we observe the immunosuppressive effects in blood (see Section 4.1). Previous results obtained in brown trout (*Salmo trutta*) [19], Siberian sturgeon (*Acipenser baerii*) [1], eastern mosquitofish (*Gambusia holbrooki*) [100], Atlantic salmon (*Salmo salar*) [33,101], and three-spined sticklebacks (*Gasterosteus aculeatus*) [102] also suggest that variation in telomere length in fish may be related to past temperature.

Since telomerase is a protein, it has its thermal optimum, which probably depends on the species and population. The tertiary and quaternary structures of proteins are altered by high temperatures, modifying a number of kinetic processes [11,81,103]. Moreover, telomerase activity is generally very sensitive and plastic, and changes in its activity are reversible [104]. These results demonstrate that the telomerase response and mitochondrial function are associated with cellular senescence and telomere length in whitefishes.

**Figure 7:**
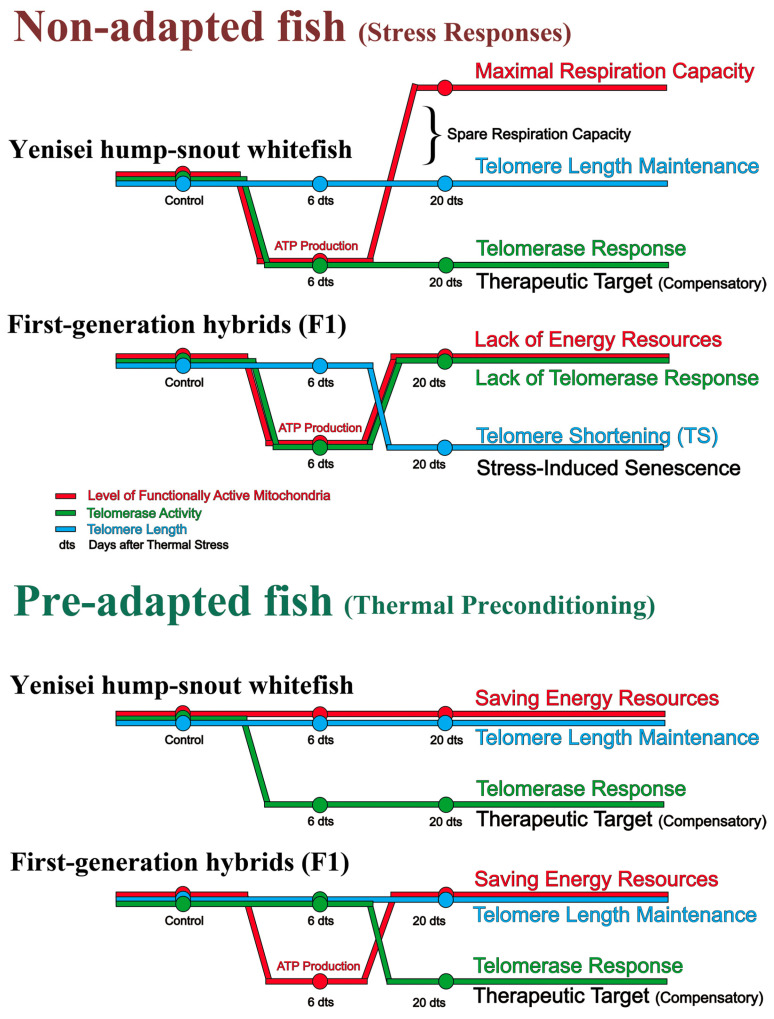
Scheme of senescence responses in non-adapted and pre-adapted hump-snout whitefish and hybrids due to acute thermal stress.

Figure 7 shows a generalized scheme of the above-listed senescence responses related to thermal stress and pre-adaptation in hump-snout whitefish and its hybrids.

### 4.3. Relationship with Other Biomarkers and Prospects for Further Research

The effects of thermal preconditioning and associated shifts in mitochondria and telomerase expression examined in this work may correlate with the responses of other biomarkers described in previous work [5,34,47,57,105]. Stress exposure in the early stages of embryogenesis could lead to physiological changes in later life stages, as shown in a number of studies in fish [5,34,47,57,105]. By monitoring plasma cortisol levels, it was possible to evaluate how preconditioning affected the overall stress response of thermally preconditioned rainbow trout (*Oncorhynchus mykiss*) in the juvenile stage. Examination of plasma cortisol levels revealed significantly lower levels in the preconditioned fish compared to the non-preconditioned control fish [57]. A number of changes for *IL-1β, IL-6, TNF-α, IFN-1, β2m, MH class I*, and *HSP70* transcripts were also induced by thermal preconditioning of juvenile fish, but the patterns varied in different tissues [57].

The lake whitefish (*Coregonus clupeaformis*) embryos also showed a plastic response at the level of HSP proteins after repeated stress [5,47]. The observed reduction in response to thermal stress in lake whitefish embryos indicates that embryos of this species are plastic. Although repeated thermal stress can protect against extreme stress, the response varies as a function of temperature and duration [47]. In other experiments, HSP protein expression profiles show that lake whitefish (*C. clupeaformis*) are physiologically more sensitive to higher temperatures than to lower temperatures [5]. In addition, zebrafish (*Danio rerio*) exposed to different temperatures during their embryonic period have also shown developmental plasticity. Scott and Johnston [105] have shown that the temperature experienced by zebrafish during their early life stages can alter their thermal acclimation, which is not fixed.

Consequently, the early environment influences many developmental processes, such as stress responses, and has the power to alter the developmental course of an organism [47,106]. Moreover, the earlier preconditioning training is used during development, the greater the expected effect [106,107]. Therefore, future research should focus on how preconditioning affects the ability of whitefish to withstand stress at different ontogenetic stages, including embryogenesis. We would also like to focus on investigating the dynamics of the biomarkers used in this work involving other indicators, such as the gene expression involved in the thermal response and epigenetic factors, such as DNA methylation and transposons—the transposable elements.

### 4.4. Understanding Whitefish Stability in the Context of their Ecology and Speciation

This work may be of interest in the context of studying the mechanisms of species resistance during whitefish speciation. This fact is due to the reason that hybridization of whitefish has an evolutionary direction, as most of the studied distant forms/species of lake whitefish have passed through a lot of the stages of hybridization [38,44,108,109,110].

In this work, the first-generation (F1) hybrids of hump-snout whitefish with Baikal whitefish, obtained by artificial hybridization and not being a natural wild form of whitefish, showed lower stability and response rate after thermal shock, but were expected to have wider limits of the reaction norm due to the possible effect of heterosis. However, the hump-snout whitefish showed great stability to heat shock, suggesting the presence of stress-buffering mechanisms. The hump-snout whitefish is a wild Baikal species that has adapted to its natural environment, including temperature fluctuations, over thousands of years (about 60,000 years) [37]. This species is linked in the natural environment to habitat conditions in the river during spawning and hatching, i.e., it experiences maximum daily and seasonal temperature fluctuations [37], and thus appears to be more stable and plastic due to natural acclimation. In addition, the artificially pre-adapted hump-snout whitefish in this experiment showed plasticity of tolerance limits due to reliable readaptation.

According to previous model genomic and theoretical work, natural wild hybrid populations between a widespread generalist and several endemic species with a small range were previously thought to have lower susceptibility to projected climatic conditions compared to pure endemic species [110]. The lower susceptibility of wild hybrids was explained by the overlap between introgressive and adaptive genomic regions, suggesting a signal of adaptive introgression and the effects of heterosis. However, in our work we compared artificial hybrid forms with potentially plastic wild whitefish species that live within very wide limits of thermopreferendum. Currently, these hybrid forms are lacking in the natural environment due to the temporally and spatially separated spawning periods of the parental forms. However, in the context of changing climatic conditions, even such hybrids whose occurrence is associated only with artificial (man-made) hybridization may become common in the natural environment in the near future.

Thus, the questions of whether genes or the environment have more to do with temperature adaptation, how artificially obtained forms of hybrids will behave in the natural environment, and whether the effect of heterosis will be observable are very important at this time. The general patterns will emerge only in the future, as many details in the model experiments are important: species characteristics, nuances of thermal stress/shock such as duration of exposure (acute or chronic), acclimation regimen, biomarkers used, delayed effects, and dynamics. In this context, work on the transgenerational effects of preconditioning in whitefish will be no less interesting.

## 5. Conclusions

The focus of this work was the study of physiological aging (senescence) of whitefish under the influence of thermal stress. Particularly clear results on the effectiveness of the use of pre-adaptation are shown by the example of hybrids of hump-snout whitefish with Baikal whitefish obtained by artificial hybridization, which are not natural wild forms of whitefish. The hybrids showed lower stability and reaction rates after thermal stress. Nevertheless, temperature pre-adaptation of such unstable hybrids appeared to be a trigger for compensatory mechanisms at the cellular and molecular levels. In contrast, as evolutionarily novel and unstable forms, non-adapted hybrids showed depletion of mitochondrial energy resources and telomeres after thermal stress.

Currently, we can speak more about the prospect of using natural stable species as model forms in aquaculture, such as the hump-snout whitefish, which naturally lives in lakes and rivers that are highly variable in terms of temperature. In particular, the results obtained confirm the promising use of hump-snout whitefish as an object for aquaculture. The pre-adapted hump-snout whitefish in this experiment demonstrate the plasticity of tolerance limits through reliable reacclimation, which can be very interesting for commercial fish farming. Hatchery-reared whitefish are released for recreational fishing or cultivated to replace wild stocks as natural populations are scarce. Therefore, thermal conditions and thermal preconditioning during hatchery rearing prepare juvenile fish of natural species for the new thermal conditions in the environment, and mitigate the effects of ongoing climate warming. Thus, preliminary temperature adaptation may serve as good technology to maintain homeostasis in farmed whitefish forms and prevent physiological aging in response to excessive temperature fluctuations.

This work is also important for modeling and understanding the mechanisms of whitefish adaptation at the molecular and cellular levels to new temperature conditions. Understanding the mechanistic basis of whitefish plasticity and thermal tolerance could help determine how different forms of whitefishes will respond in the future to changes in the natural environment due to climate warming.

## Figures and Tables

**Table 1:** Characteristics of the whitefishes used in the study.

		Average Length, TL, cm	Weight, g	Number of Fish	Number of Samples
Hump-snout whitefish (control and experiment)	6 d	6.31 ± 0.43	3.80 ± 0.48	28	224
	20 d	7.57 ± 1.70	5.83 ± 1.60	28	224
Hybrids (control and experiment)	6 d	5.94 ± 0.75	2.32 ± 0.94	28	224
	20 d	6.60 ± 0.62	3.47 ± 0.92	28	224

**Table 2:** Dissolved oxygen and carbon dioxide concentrations in the aquariums.

Aquariums	Dissolved Oxygen, mg/L	Saturation O_2_, %	CO_2_, mg/L
Control, 17 °C	9.9	107	2.4
Training, 21 °C	8.6	105	2.6
Experimental, 26 °C	7.8	102	2.1

## Appendix A

**Table A1:** Comparison of whitefish peripheral blood profiles ^1^ (percentage of the total blood cells) in the control and following 6 and 20 days of thermal exposure.

				EB ^1^	MRBC ^1^	LC ^1^	MC ^1^	INP ^1^	PMNP ^1^	MP ^ 1 ^	N:L ^1^
Non-adapted Hump-snout whitefish	Experiment	6d	M	16.8	83.2	61.6	3.9 ***↑	18.6 ***↓	15.9 ***↑	0	0.56 ***↓
			SD	1.8	1.8	8.6	1.2	3.6	2.3		0.04
		20d	M	13.5	86.5	95.3	0	0 ***↓	4.8 ***↑	0	0.05 ***↓
			SD	1.2	1.2	8.3			0.2		0.01
	Control	6d	M	15.5	84.5	57.1	0	42.9	0	0	0.75
			SD	2.3	2.3	5.8		1.7			0.02
		20d	M	16.6	83.4	81.1	0	18.9	0	0	0.23
			SD	1.9	1.9	8.1		3.1			0.01
Pre-adapted Hump-snout whitefish											
	Experiment	6d	M	24.1 *↑	75.9 *↓	80 *↑	0	17.5 ***↓	2.5	0	0.25 ***↓
			SD	23.8	2.8	7.8		2.4	0.3		0.01
		20d	M	14.2	85.8	72.7 *↓	0	17.8	9.5 ***↑	0	0.38 **↑
			SD	1.6	1.6	7.9		0.39	0.8		0.04
	Control	6d	M	15.5	84.5	57.1	0	42.9	0	0	0.75
			SD	2.3	2.3	5.8		1.7			0.02
		20d	M	18.8	81.2	81.1	0	18.9	0	0	0.23
			SD	1.7	1.7	3.1		1.1			0.03
Non-adapted Hybrids											
	Experiment	6d	M	25 ***↑	75 ***↓	79.9 ***↑	0 ***↓	20.1	0 ***↓	0	0.25 ***↓
			SD	2.7	2.7	7.8		3.6			0.01
		20d	M	14.6	85.4	66.7	0	5.6 **↓	27.7	0 ***↓	0.5
			SD	1.9	1.9	7.4		1.1	3.1		0.06
	Control	6d	M	7.8	92.2	58.8	5,9	23.5	11.8	0	0.6
			SD	1.8	1.8	3.2	1.2	3.6	0.7		0.04
		20d	M	15.7	84.3	62	0	16	20	2	0.58
			SD	1.7	1.7	7.2		2.9	1.1	0.07	0.06
Pre-adapted Hybrids											
	Experiment	6d	M	20.6 ***↑	79.4 ***↓	67.6	2.7	10.8 **↓	18.9 **↑	0	0.44 **↓
			SD	2.4	2.3	8.6	0.9	2.2	2.3		0.03
		20d	M	12.1	87.9	67.1 *↑	1.4 ***↑	25.8	1.2 ***↓	5.5 ***↓	0.4 **↓
			SD	1.9	1.9	12.1	0.3	3.4	0.08	0.9	0.03
	Control	6d	M	7.8	92.2	58.8	5.9	23.5	11.8	0	0.6
			SD	1.8	1.8	3.2	1.2	3.6	0.7		0.04
		20d	M	16.8	83.2	36.5	0	34.5	17.5	14.5	1.42
			SD	1.6	1.6	0.73		2.1	0.9	0.7	0.32

^1^ Red blood cells: EB—Erythroblasts, MRBC—Mature red blood cells; White blood cells: LC—Lymphocytes, MC—Monocytes, INP—Immature neutrophils, PMNP—Polymorphonuclear neutrophils, MP—Macrophages, N:L—Neutrophils to lymphocytes ratio. The total lymphoblasts and lymphocytes are included to lymphocytes. In the data, mean (M) ± standard deviation (SD) is displayed. Asterisks denote statistically significant differences between the experimental and control groups: * *p* < 0.05, ** *p* < 0.01, *** *p* < 0.001 (Kruskal–Wallis test). Arrows show a significant decrease (↓) or increase (↑) in the parameters.

**Table A2:** Cytometric parameters ^1^ of whitefish red blood cells in the control and following 6 and 20 days of thermal exposure.

			E ^1^	S ^1^	s ^1^	NCR ^1^	E ^1^	S (μK^2^)^1^	s (μK^2^) ^1^	NCR ^1^
		Control					Experiment			
		Non-adapted Fish								
Hump-snout whitefish	6 d	M	0.59	213.16	49.76	0.31(140)	0.59	218.82	49.53	0.30 (260)
		SD	0.01	4.39	0.75	0.01	0.00	3.04	0.65	0.01
	20 d	M	0.61	277.25	57.93	0.27 (346)	0.60	249.75 ***↓	62.80 ***↑	0.35 ***↑ (204)
		SD	0.01	2.78	0.56	0.00	0.01	3.95	0.62	0.01
		Pre-adapted fish								
	6 d	M	0.59	213.16	49.76	0.31 (140)	0.58	206.84	52.38	0.35 **↑ (160)
		SD	0.01	4.39	0.75	0.01	0.01	3.79	1.10	0.01
	20 d	M	0.64	281.61	70.39	0.36 (156)	0.55 ***↓	230.58 ***↓	52.08 ***↓	0.29 *↓ (141)
		SD	0.01	11.49	5.35	0.03	0.01	3.09	0.79	0.01
Hybrids		Non-adapted fish								
	6 d	M	0.63	224.51	50.35	0.30 (165)	0.61	251.13 ***↑	44.82 ***↓	0.22 ***↓ (139)
		SD	0.01	3.88	0.66	0.01	0.01	4.84	1.22	0.01
	20 d	M	0.61	253.03	59.42	0.33 (176)	0.58 **↓	245.70	50.13 ***↓	0.27 ***↓ (394)
		SD	0.01	5.64	0.80	0.01	0.01	4.04	0.68	0.01
		Pre-adapted fish								
	6 d	M	0.63	224.51	50.35	0.30 (165)	0.60	250.45 ***↓	60.21 ***↑	0.33 (155)
		SD	0.01	3.88	0.66	0.01	0.01	5.72	1.23	0.01
	20 d	M	0.57	230.2	46.21	0.26 (160)	0.59 ***↑	243.1 **↑	51.2 ***↑	0.28 (212)
		SD	0.01	3.33	0.55	0.01	0.004	2.37	0.44	0.005

^1^ E—Eccentricity, S (μK2)—Cell Area, s (μK2)—Nuclear Area, NCR—nuclear-cytoplasmic ratio (the number of analyzed cells is given in parentheses next to the NCR value). The number of studied cells is indicated in brackets. In the data, mean (M) ± standard deviation (SD) is displayed. Asterisks denote statistically significant differences between the experimental and control groups: * *p* < 0.05, ** *p* < 0.01, *** *p* < 0.001 (Kruskal–Wallis test). Arrows show a significant decrease (↓) or increase (↑) in the parameters.

**Table A3:** Comparison of whitefish blood profiles ^1^ in the hematopoietic organs (kidneys) (percentage of the total blood cells) in the control and following 6 and 20 days of thermal exposure.

			BL ^1^	PH ^1^	MCB ^1^	LB ^1^	PC ^1^	BL ^1^	PH ^1^	MCB ^1^	LB ^1^	PC ^1^
			Control					Experiment				
		Non-adapted Fish										
Hump-snout whitefish	6 d	M	12.2	14.2	4.1	60.8	8.7	10.3	13.65	1.05 ***↓	71.6	3.4 ***↓
		SD	0.9	0.3	0.3	2.3	0.8	1.2	0.8	0.07	4.1	0.7
	20 d	M	7.2	14.9	4.2	33.65	12.45	7.65 ***↑	26.45 ***↑	6.6 **↑	46.05 *↑	13.25
		SD	0.8	0.8	0.3	2.5	1.9	0.8	0.8	0.7	4.2	0.9
		Pre-adapted fish										
	6 d	M	12.2	14.2	4.1	60.8	8.7	12.2	21.45 ***↑	1.85 ***↓	61.1	3.4 ***↓
		SD	0.9	0.3	0.3	2.3	0.8	0.9	1.3	0.2	3.7	0.2
	20 d	M	12.7	17.6	0.6	60.4	8.7	7 **↓	15.5	0.45 ***↓	70.15 *↑	6.9
		SD	1.4	1.1	0.02	3.1	0.8	1.2	2.1	0.03	3.9	0.9
		Non-adapted fish										
Hybrids	6 d	M	14.3	18.5	2.45	38.8	25.95	17.9	8.05 ***↓	0 ***↓	67 ***↑	7.05 ***↓
		SD	1.3	0.9	0.09	2.1	3	1.9	0.7	0	7.3	0.9
	20 d	M	7.8	16.5	1.1	67.8	6.8	7.15	11.5 **↓	1.95 ***↑	51.7	27.7 ***↑
		SD	0.9	0.7	0.05	3.4	0.3	1.5	1.7	0.08	9.2	6.2
		Pre-adapted fish										
	6 d	M	14.3	18.5	2.45	38.8	25.95	14.65	15.65	1.4 ***↓	63.15 **↑	5.15 ***↓
		SD	1.3	0.9	0.09	2.1	3	2.1	1.2	0.07	7.1	0.9
	20 d	M	5.85	52.5	2.9	38.75	0	9.3 **↑	18.25 ***↓	6.7 ***↑	39.8	13.8 ***↑
		SD	0.8	6.8	0.1	2.3	0	0.9	2.4	0.23	8.1	0.9

^1^ BL—Blasts, PC—Phagocytes, MCB—Monocytoblasts, LB—Lymphoblasts, PC—Plasmocytes. In the data, mean (M) ± standard deviation (SD) is displayed. Asterisks denote statistically significant differences between the experimental and control groups: * *p* < 0.05, ** *p* < 0.01, *** *p* < 0.001 (Kruskal–Wallis test). Arrows show a significant decrease (↓) or increase (↑) in the parameters.

**Table A4:** Comparison of whitefish blood profiles ^1^ in the hematopoietic organs (spleen) (percentage of the total blood cells) in the control and following 6 and 20 days of thermal exposure.

			BL ^1^	PH ^1^	MCB ^1^	LB ^1^	PC ^1^	BL ^1^	PH ^1^	MCB ^1^	LB ^1^	PC ^1^
			Control					Experiment				
		Non-adapted Fish										
Hump-snout whitefish	6 d	M	5.8	36.6	11.85	38.3	7.45	2.95 **↓	23.05 ***↓	0 ***↓	74 *↑	0 **↓
		SD	0.9	2.4	0.9	3.3	0.8	0.06	1.3	0	11	0
	20 d	M	6.5	41	10	35	7.5	6.55	33.15	20 ***↑	37.8	2.5 ***↓
		SD	0.8	3.6	0.9	1.6	0.8	0.7	2.2	1.1	2.4	0.06
		Pre-adapted fish										
	6 d	M	5.8	36.6	11.85	38.3	7.45	9.8 ***↑	20.3 ***↓	2.25 ***↓	66.05 **↑	1.6 ***↓
		SD	0.9	2.4	0.9	3.3	0.8	0.7	0.9	0.01	9.2	0.06
	20 d	M	7.2	45	8	33	6.8	6.55	33.15	2.5 ***↓	37.8	2.5 ***↓
		SD	1.3	7.2	0.7	0	0	0.7	1.4	0.02	7.1	0.06
		Non-adapted fish										
Hybrids	6 d	M	4	31.9	3.25	60.1	0.75	2.75	51.5 ***↑	2.35 *↓	43.1 *↓	0.3 ***↓
		SD	0.8	3.1	0.4	6.9	0.01	0.03	4.8	0.08	3.8	0
	20 d	M	0	47.7	2.15	46.8	4.35	0	63.6	1.2 ***↓	24.4 *↓	10.8 ***↑
		SD	0	7.4	0.07	6.9	0.9	0	7.5	0.08	5.3	0
		Pre-adapted fish										
	6 d	M	4	18.5	31.9	60.1	0.75	7	58 ***↑	5***↓	25 ***↓	5 ***↑
		SD	0.8	0.9	3.1	6.9	0.01	1.8	14.5	0.9	6.3	0.7
	20 d	M	0	52.5	46.7	46.8	4.35	6.2 ***↑	51.6	5.1 **↓	33 ***↓	4.1
		SD	0	6.8	4.7	5.8	0.12	1.1	11.8	0.9	8.2	0.8

^1^ BL—Blasts, PC—Phagocytes, MCB—Monocytoblasts, LB—Lymphoblasts, PC—Plasmocytes. In the data, mean (M) ± standard deviation (SD) is displayed. Asterisks denote statistically significant differences between the experimental and control groups: * *p* < 0.05, ** *p* < 0.01, *** *p* < 0.001 (Kruskal–Wallis test). Arrows show a significant decrease (↓) or increase (↑) in the parameters.

**Table A5:** Relative telomerase activity (RTA) in different whitefish tissues.

Hump-snout whitefish			Non-adapted Fish			
			Gills		Liver	
			Control	Experiment	Control	Experiment
	6 d	M	1.44	0.92	3.68	0.34 ***↓
		SD	0.72	0.5	0.79	0.22
	20 d	M	2.71	3	3.69	1.22 **↓
		SD	0.63	0.68	0.64	0.65
			Pre-adapted fish			
			Control	Experiment	Control	Experiment
	6 d	M	1.44	1.45	3.68	0.77 **↓
		SD	0.72	0.53	0.79	0.51
	20 d	M	4.27	6.81	10.32	7.12 *↓
		SD	1.09	3.5	2.52	2.5
Hybrids			Non-adapted fish			
			Gills		Liver	
			Control	Experiment	Control	Experiment
	6 d	M	1.9	0.59 **↓	1.1	0.78
		SD	0.50	0.24	0.45	0.39
	20 d	M	2.99	2.86	1.5	1.73
		SD	0.64	0.69	0.69	0.44
			Pre-adapted fish			
			Control	Experiment	Control	Experiment
	6 d	M	1.9	1.38	1.1	1.29
		SD	0.50	0.78	0.45	0.35
	20 d	M	12.42	6.58 *↓	5.5	7.98
		SD	2.11	2.97	1.65	4.53

In the data, mean (M) ± standard deviation (SD) is displayed. Asterisks denote statistically significant differences between the experimental and control groups: * *p* < 0.05, ** *p* < 0.01, *** *p* < 0.001 (Kruskal–Wallis test). Arrows show a significant decrease (↓) in the parameters.

**Table A6:** Relative telomere length (RTL) in different whitefish tissues.

Hump-snout whitefish		Non-adapted Fish				
			Gills		Liver	
			Control	Experiment	Control	Experiment
	6 d	M	1.11	1.22	1.00	1.10
		SD	0.14	0.14	0.15	0.13
	20 d	M	1.17	1.12	1.19	1.16
		SD	0.14	0.26	0.15	0.18
		Pre-adapted fish				
			Control	Experiment	Control	Experiment
	6 d	M	1.11	1.34	1.00	0.94
		SD	0.14	0.27	0.15	0.08
	20 d	M	0.84	0.84	0.94	0.97
		SD	0.09	0.15	0.16	0.10
Hybrids		Non-adapted fish				
			Gills		Liver	
			Control	Experiment	Control	Experiment
	6 d	M	0.91	0.92	0.97	0.97
		SD	0.09	0.12	0.16	0.22
	20 d	M	1.16	0.85	1.16	1.24
		SD	0.24	0.22 *↓	0.24	0.09
		Pre-adapted fish				
			Control	Experiment	Control	Experiment
	6 d	M	0.91	0.89	0.97	0.87
		SD	0.09	0.14	0.16	0.08
	20 d	M	1.09	1.04	0.84	0.85
		SD	0.25	1.14	1.14	0.22

In the data, mean (M) ± standard deviation (SD) is displayed. An asterisk denotes statistically significant differences between the experimental and control groups: * *p* < 0.05 (Kruskal–Wallis test). Arrow shows a significant decrease (↓) in the parameters.
